# Inhibition of myostatin and related signaling pathways for the treatment of muscle atrophy in motor neuron diseases

**DOI:** 10.1007/s00018-022-04408-w

**Published:** 2022-06-21

**Authors:** Elena Abati, Arianna Manini, Giacomo Pietro Comi, Stefania Corti

**Affiliations:** 1 Department of Pathophysiology and Transplantation (DEPT), Dino Ferrari Centre, Neuroscience Section, Neurology Unit, Fondazione IRCCS Ca’ Granda-Ospedale Maggiore Policlinico, University of Milan, Milan, Italy; 2 Neurology Unit, Department of Neuroscience, Fondazione IRCCS Ca’ Granda Ospedale Maggiore Policlinico, Milan, Italy; 3 Neuromuscular and Rare Diseases Unit, Department of Neuroscience, Fondazione IRCCS Ca’ Granda Ospedale Maggiore Policlinico, Milan, Italy

**Keywords:** Myostatin, Motor neuron diseases, Muscle atrophy, Activin receptors, type II, Monoclonal antibodies

## Abstract

Myostatin is a negative regulator of skeletal muscle growth secreted by skeletal myocytes. In the past years, myostatin inhibition sparked interest among the scientific community for its potential to enhance muscle growth and to reduce, or even prevent, muscle atrophy. These characteristics make it a promising target for the treatment of muscle atrophy in motor neuron diseases, namely, amyotrophic lateral sclerosis (ALS) and spinal muscular atrophy (SMA), which are rare neurological diseases, whereby the degeneration of motor neurons leads to progressive muscle loss and paralysis. These diseases carry a huge burden of morbidity and mortality but, despite this unfavorable scenario, several therapeutic advancements have been made in the past years. Indeed, a number of different curative therapies for SMA have been approved, leading to a revolution in the life expectancy and outcomes of SMA patients. Similarly, tofersen, an antisense oligonucleotide, is now undergoing clinical trial phase for use in ALS patients carrying the SOD1 mutation. However, these therapies are not able to completely halt or reverse progression of muscle damage. Recently, a trial evaluating apitegromab, a myostatin inhibitor, in SMA patients was started, following positive results from preclinical studies. In this context, myostatin inhibition could represent a useful strategy to tackle motor symptoms in these patients. The aim of this review is to describe the myostatin pathway and its role in motor neuron diseases, and to summarize and critically discuss preclinical and clinical studies of myostatin inhibitors in SMA and ALS. Then, we will highlight promises and pitfalls related to the use of myostatin inhibitors in the human setting, to aid the scientific community in the development of future clinical trials.

## Introduction

Motor neuron diseases (MND) are a group of progressive neurodegenerative disorders which selectively affect the cellular population of motor neurons (MNs) [1, 2]. MN are localized either in the cortex (upper MNs) or in the brainstem and anterior horns of the spinal cord (lower MNs). The two most common and widely known MNDs are amyotrophic lateral sclerosis (ALS) and spinal muscular atrophy (SMA), which differ for pathogenic mechanisms, age at onset and presence of upper MN involvement [1, 2].

ALS is a fatal disorder that targets both upper and lower MNs, causing progressive weakness and atrophy of skeletal muscles, which usually leads to paralysis and death within 3–5 years [2]. ALS is divided in sporadic (sALS), when occurring in absence of family history, and familial (fALS), when at least two other family members are affected. sALS represents 85–90% of all cases and presents a later age of onset (58–63 years), while fALS accounts for the remaining 10–15% of cases and shows a slightly younger age of onset (47–53 years) [2, 3]. Potential causative mutations have been described in over 50 genes. Among them, *C9orf72, TARDBP, FUS* and *SOD1* account for almost 75% of fALS cases [2, 3]. As for now, the pathogenic mechanisms leading to ALS development have not been completely clarified. Because of our limited knowledge, our arsenal is also devoid of efficient treatments, even though new therapeutic strategies, such as gene silencing and regenerative therapies, might prove useful [4, 5]. Notably, tofersen, an antisense oligonucleotide, recently proved effective in a clinical trial on ALS patients carrying a mutation in the *SOD1* gene [6].

SMA is a genetic neuromuscular disorder caused by loss-of-function mutations in the Survival Motor Neuron 1 (*SMN1*) gene, which are inherited in an autosomal recessive fashion [1]. As a consequence, the levels of the Survival Motor Neuron (SMN) protein, which is necessary for motor neuron survival in the lower brainstem and spinal cord, are severely affected, thus resulting in widespread muscle atrophy and death due to respiratory distress [1]. Indeed, SMA has represented the main genetic cause of mortality within infants during the last decades [1]. The SMN protein is ubiquitously expressed and takes part in several pathways involved in cellular homeostasis, including the assembly of the spliceosomal machinery, endocytosis, and protein translation [7]. Therefore, *SMN* loss of function can impact multiple systems beyond MNs [8–10].

The genome sequence of *SMN2*, the paralogous gene of *SMN1*, resembles that of *SMN1* except for the presence of a thymine instead of a cytosine at codon 280 in exon 7 [11]. This results in the exclusion of exon 7 during splicing from 85 to 90% of transcripts, which encode a truncated, non-functional SMN protein [12]. The remaining 10–15% of *SMN2*-derived mRNA is translated into a functional SMN protein, whose low levels partly compensate for the loss of *SMN1*-derived products in patients affected by SMA [11]. In this scenario, the clinical heterogeneity of SMA patients derives from the variable number of *SMN2* copies found in the general population [13]. According to the severity of the clinical picture and to the age of onset, SMA is classified in five subtypes—0,1,2,3,4 [14–16]. Type 0 SMA has prenatal onset and is usually fatal in utero [16]. The most common form is represented by type 1, which appears before 6 months of age. Patients are unable to sit without support and, if untreated, die within 2 years of age, typically due to respiratory insufficiency [14]. The onset of type 2 SMA is usually in early childhood and it leads to progressive proximal muscle weakness, inability to walk, scoliosis, tendon retractions and restrictive lung disease [16]. Type 3 SMA usually starts later in childhood and causes similar symptoms, albeit less severe, which may in some cases lead to the loss of the ability to walk [16]. Type 4 SMA is the mildest form of the disease and manifests itself during adult life [16]. In the past years, three innovative therapies for SMA, namely, nusinersen, an antisense oligonucleotide, onasemnogene abeparvovec, an adeno-associated virus (AAV)-mediated gene therapy, and risdiplam, an orally delivered splicing modifier small-molecule, have been approved by the Food and Drug Administration (FDA) and European Medicine Agency (EMA), and have allowed the achievement of substantial improvements in survival and motor performance of SMA patients [17–19].

Despite the previously inconceivable results obtained by these revolutionary therapies, additional treatments aimed at mitigating the functional impact of these diseases and, therefore, ameliorating the quality of life of SMA and ALS patients are warranted. The interest of the scientific community has recently focused on treatments able to reduce or even prevent muscle atrophy, or otherwise to enhance muscle growth, including inhibitors of myostatin (growth differentiation Factor 8; GDF-8), a member of the transforming growth factor-β (TGFβ) superfamily which acts as negative regulator of muscle mass. The aim of this review is to describe the myostatin pathway and its role in MNDs, and to summarize and critically discuss preclinical and clinical studies of myostatin inhibitors in SMA and ALS. Then, we will highlight the most relevant issues related to the use of myostatin inhibitors in the human setting, to aid the scientific community in the development of future clinical trials.

## Myostatin pathways and their role in MNDs

### Biology of myostatin

Myostatin is a paracrine signaling molecule identified in 1997, that belongs to the TGFβ superfamily. It is mainly secreted by skeletal myocytes, and negatively regulates skeletal muscle growth through activin receptors [20]. It is encoded by the *MSTN* gene, whose amino acid sequence is strongly conserved in evolution [21]. Engineered or naturally occurring mutations in the *MSTN* gene in human and animal species determine an increase in muscle mass, with higher quantity and size of myofibers, in absence of an increment in cell proliferation [22–29]. During the embryological phase, myostatin is expressed in the myotome compartment of developing somites [30, 31]. In adult animals, the expression of myostatin remains restricted to the skeletal muscles, albeit lower levels of myostatin RNA have also been detected in the adipose tissue as well [20].

**Fig. 1:**
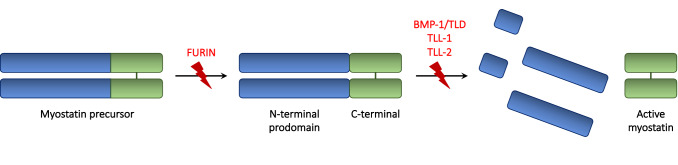
Schematic representation of myostatin processing. Promyostatin, the inactive precursor of MSTN, is composed by the N-terminal prodomain and the C-terminal dimer. Myostatin activation requires two enzymatic cleavages, operated by the furin proteases and by the BMP/tolloid metalloproteases, respectively, including the bone morphogenetic protein-1/tolloid (BMP-1/TLD), tolloid-like-1 (TLL-1) and tolloid-like-2 (TLL-2). In the latent myostatin, resulted by furin cleavage, the non-covalent binding between the C-terminal and the prodomain prevents myostatin activation. Subsequently, the tolloid cleavage at the aspartate residue 76 allows the release and activation of myostatin

**Fig. 2:**
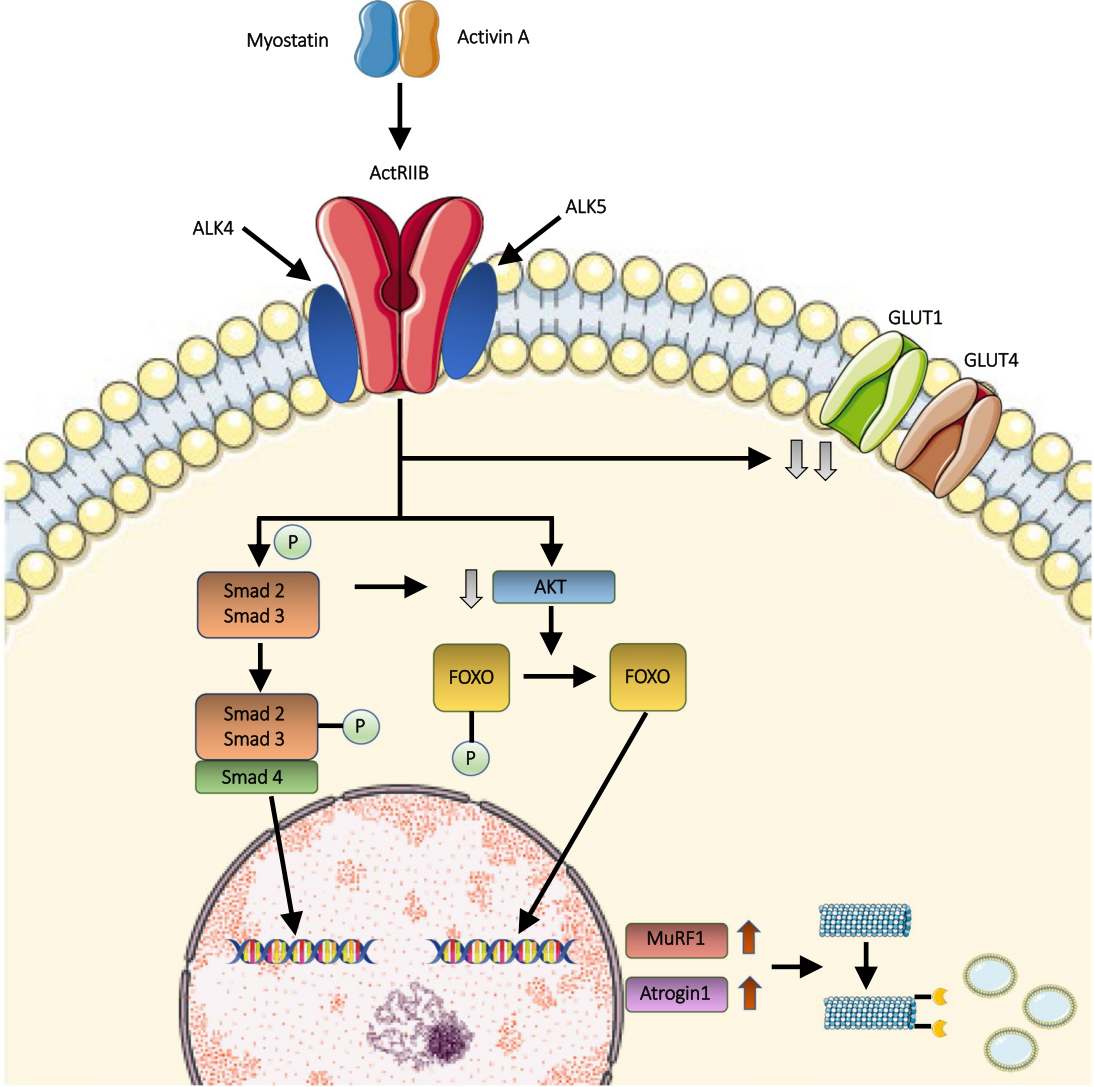
Myostatin muscle pathway. The binding of myostatin or, alternatively, activin, to muscle activin receptor type IIB (ActRIIB) results in its dimerization and, subsequently, in the activation of type I activin receptor transmembrane kinases ALK4 or ALK5. Consequently, the Smad2/Smad3 complex is phosphorylated, and the Smad4 component is recruited. The Smad complex enters the nucleus, where it acts as transcriptional activator of downstream genes involved in muscle wasting. Furthermore, the activation of the transmembrane activin receptor leads to the downregulation of AKT, which is involved in FOXO phosphorylation. Dephosphorylated FOXO translocates into the nucleus, and up-regulates the transcription of MuRF1 and Atrogin1. Muscle proteins, which are ubiquitinated by MuRF1 and Atrogin1, are subsequently catabolized by proteasomes, thus resulting in muscle atrophy. Simultaneously, myostatin is involved in glucose homeostasis regulation, likely by reducing the protein levels of glucose transporter 1 (GLUT1) and 4 (GLUT4)

Myostatin is secreted as promyostatin, its inactive precursor containing a prodomain which prevents binding of mature myostatin peptides [32, 33] (Fig. 1). The conversion of promyostatin in the active form occurs in two steps [34, 35]. The first cleavage is carried on by a proprotein convertase (furin protease), and results in the formation of latent myostatin (Fig. 1). Latent myostatin is kept inactive by the presence of a noncovalent binding between the prodomain (N-terminus) and the mature myostatin (C-terminus) [34, 35] (Fig. 1). Next, this complex undergoes a second cleavage by the bone morphogenetic protein-1/tolloid (BMP-1/TLD), tolloid-like-1 (TLL-1) and tolloid-like-2 (TLL-2) proteases, which release mature myostatin dimers [36] (Fig. 1). The majority of circulating myostatin is found in its bound, inactive form [37, 38]. Notably, the isolated propeptide is capable of halting myostatin activity both in vitro and in vivo [37]. At the extracellular level, myostatin is regulated by several other binding proteins. Among these, follistatin is capable of binding multiple TGF-β family members, including myostatin, thus preventing its pairing with receptors [39–44]. Studies in mice support a key role of follistatin in modulating myostatin activity in vivo [35]. Myostatin can be negatively regulated by other molecules including GDF-associated serum protein (GASP)-1, GASP-2, follistatin-like (FSTL)-3, and latent TGF-β-binding protein (LTBP)-3. The active form of myostatin exerts its action through the two activin type II receptors (ActRIIA and ActRIIB) [35]. The binding of myostatin to ActRIIA and ActRIIB leads to the engagement of active activin-like kinases (ALK) 4/5 [35] (Fig. 2). This results in the phosphorylation of the Smad2/Smad3 complex and, consequently, in the recruitment of the Smad4 component [45]. Simultaneously, the activation of the transmembrane activin receptor results in the downregulation of AKT (directly and indirectly via Smad), and, downstream, in the phosphorylation of FOXO [46]. After entering the nucleus, both the Smad complex and FOXO act as transcriptional activators of downstream genes involved in muscle wasting, including MuRF1 and Atrogin1 [47]. The ubiquitination of muscle proteins mediated by MuRF1 and Atrogin1 accelerates their catabolism in the proteasomes, thus resulting in muscle atrophy [45, 47, 48] Notably, several lines of evidence suggest the existence of other TGF-β family members with similar functions compared to myostatin [39, 49–52]. Among those, activin A is a key ligand that works with myostatin to restrict muscle growth [49, 52]. Other putatively involved molecules include known (such as GDF11) and unknown members of the TGF-β superfamily [53]. GDF11 and myostatin are closely related TGF-β superfamily proteins with a significant degree of homology in their sequence [54]. GDF-11 plays several physiological roles, being involved not only in the regulation of muscle homeostasis and mass but also in aging and in protection of cardiac tissue from stress and disease [55].

Some studies suggest that there might be an association between age and sex and circulating levels of myostatin, follistatin and other TGF-β family members, such as activin A and GDF11. Age seems to be positively associated with an increase in myostatin levels in women, with higher levels than old male counterparts, while myostatin levels are higher in young men in comparison with young women [56]. Another study confirmed that, in a sample of people aged 60 or older, women had higher plasma levels of myostatin and GDF11 than men, while men had higher plasma levels of follistatin than women [57]. Conversely, another study found that GDF11 levels do not decline throughout aging and do not statistically differ between sexes in healthy adults [55]. This difference might be due to the use of different measurement techniques which may not be able to effectively discriminate between GDF11 and myostatin because of their sequence homology [55]. In addition, increased serum concentration of activin A were found to be associated with age in both men and postmenopausal women [58]. A relationship between myostatin levels and sexual hormones have been reported, even though studies conducted so far yielded conflicting results [59–61]. A better understanding of these determinants is needed to elucidate a potential confounding effect of these variables on the results of clinical trials.

### The role of myostatin in the regulation of muscle homeostasis

The skeletal muscle is a dynamic tissue that continuously renews during adult life. A pool of muscle stem cells, also called satellite cells, is present within muscles during all life stages [62, 63]. Satellite cells are usually quiescent but can activate after muscle injury, differentiating into myoblasts and myocytes [45]. Myocytes then form syncytia—or myotubes—which can become both fast and slow twitch muscle fibers [62]. Cells at different stages can coexist within a muscle at the same time, and tissue homeostasis is maintained through the tight regulation of cell type distribution and quantity. Myostatin release increases progressively during the development, and developed cells inhibit satellite cells proliferation and differentiation through a feedback loop mechanism [45]. Indeed, myogenic stem cells and proliferating myoblasts express ActRIIB and ALK 4/5 receptors. Following myostatin activation and binding, these receptors trigger a downstream cascade that results in the inhibition of the muscle regulatory factors (MRFs) [64]. MRFs are a set of regulatory factors, namely, Myf5, MyoD, MRF4 and myogenin, which drive muscle cell development and whose inhibition is capable of blocking satellite cell activation, stopping cell cycle progression in proliferating myoblasts and interrupting myogenesis [64–67]. Since MRFs also promote myostatin transcription, the myostatin cascade auto-regulates itself by repressing its own transcription [64]. In differentiated cells, myostatin prolongs the overall survival by reducing the rates of apoptosis via the p53 pathway [64, 68]. Taken together, these findings point out that myostatin limits muscle growth by reducing myogenesis rather than promoting muscle cell apoptosis.

### The role of myostatin in metabolic homeostasis

In addition to its role in maintaining muscle homeostasis, myostatin deficiency was shown to have beneficial effects on systemic metabolic and glucose homeostasis regulation. This improvement is likely secondary to the activation of anabolic processes in muscles, such as increased insulin-stimulated skeletal muscle glucose uptake through glucose transporter 1 (GLUT1) and 4 (GLUT4) (Fig. 2) [69–72]. At a molecular level, some authors postulated that this could result from ActRIIA/B activation. This hypothesis is supported by the observation that selective myofiber knockout of these receptors or, alternatively, mice treatment with receptor blockade not only induced a dramatic increase in muscle mass, but also a significant decrease of fat mass and improved glucose control [73–75].

Conversely, according to other authors, these effects might have been produced by inhibition of direct signaling to other cell types, especially brown adipose tissue (BAT) cells [76]. Interestingly, a study conducted by Fournier and colleagues revealed that the ActRIIB pathway is also a negative regulator of BAT, which is a key tissue in the regulation of energy expenditure [77]. Indeed, it was observed that myostatin inhibition in mice led to increased basal metabolic rate [78]. Thus, ActRIIB blockade may exert its beneficial metabolic action also by enhancing thermogenesis and energy consumption via the BAT.

Furthermore, myostatin might play a role in adipogenesis. In *MSTN*−/− mice the amount of fat tissue was limited, thus possibly suggesting a pro-adipogenic effect of myostatin [72]. In addition to that, an in vitro study revealed that treatment of C3H10T1/2 mesenchymal stem cells with recombinant myostatin induced adipogenesis and inhibited myogenesis [79]. However, another study reported quite the opposite, stating that myostatin was able to block BMP7-induced adipogenesis in both the same mesenchymal precursor cells and preadipocytes [80]. These findings might appear contradictory at first. However, this discrepancy might be explained by various effects of myostatin on different cell lineages. In this scenario, myostatin might have a pro-adipogenic or anti-adipogenic effect in presence of certain co-signals (such as BMP7). Nonetheless, further studies are needed to better elucidate the mechanisms by which myostatin influences human metabolism and body composition.

### The role of myostatin in MNDs

Evidence related to myostatin pathway activation in patients with MND is scarce. Transcriptome studies on skeletal muscle of ALS patients revealed an overexpression of the follistatin gene compared to both controls and patients with multifocal motor neuropathy [81]. However, subsequent studies on the muscles of ALS patients and of *SOD1* mouse models failed to retrieve overexpression of this pathway [82–84]. In the quest for biomarkers, another group measured serum levels of myostatin and follistatin in the serum of ALS patients with either bulbar or spinal onset [85]. They found that myostatin/follistatin ratio was significantly higher in ALS than in controls and in bulbar versus spinal ALS. Interestingly, bulbar ALS patients presented higher degree of muscle atrophy than spinal ALS at muscle fiber morphometric analysis. Moving on to SMA patients, one study analyzed serum samples from 4 SMA patients and found a dramatic increase in the levels of circulating GDF-11, a decrease in the levels of circulating myostatin and a slight increase in circulating follistatin compared to patients with other neuromuscular diseases and controls [86]. Normalising myostatin levels against myosin light chain 3 (MLC3) did not alter the results. Another study investigating the transcriptome and proteome of skeletal muscle of the severe *Smn–/–;SMN2* mouse model did not detect alterations in this pathway [87].

These studies raise two issues. The first one revolves around the significance of these findings in the context of patients with severely reduced muscle tissue. Some authors advocate that the reduction of serum myostatin in patients with muscle-atrophying diseases likely reflects the loss of muscle tissue, the main producer of myostatin [88]. Conversely, other authors disagree with this hypothesis. For instance, Mariot and colleagues pointed out that both mRNA expression in muscle tissue and protein serum levels of myostatin, follistatin, GDF11 and activin A were dysregulated in neuromuscular patients [86]. They argued that, in this perspective, reduced circulating myostatin levels might result from an altered regulation of the myostatin pathway rather than directly from muscle loss. These data indicate that myostatin might be intrinsically down-regulated in MNDs and other neuromuscular diseases, where the wasting process is established [86]. Further research is needed to confirm whether this hypothesis is correct and how such a process could influence the effect of anti-myostatin drugs.

The second question stems from the observation that the alterations in the myostatin pathway of MND patients are not reproduced in mouse models. This discrepancy might be due to a variety of reasons, for example the low number of studies. A more worrisome option is that available animal models might not precisely reproduce the mechanisms underlying muscle loss and atrophy in MND patients. Surely, this aspect warrants further research and consideration, especially when testing drugs for clinical translation. In fact, researchers should take into consideration the possibility that drug failure in these models may result from a divergence in molecular mechanisms of muscle atrophy rather than from the drug being unfit for the intended purpose. In the following paragraphs, we will introduce and discuss results of preclinical and clinical trials in MND models and patients, to highlight promises and pitfalls of this therapeutic strategy.

## Preclinical trials of myostatin inhibition in MNDs

### Spinal muscular atrophy (SMA)

The role of myostatin in the muscle atrophy and muscle wasting-related pathways makes it a promising target for the treatment of SMA, which is characterized by profound weakness and loss of muscle tissue. So far, preclinical studies conducted in rodent models explored both direct myostatin inhibition and myostatin antagonism with other molecules, such as follistatin, a natural myostatin antagonist, and follistatin analogues. In one study, researchers administered recombinant follistatin to SMN Delta7 mice. [89] Treated mice displayed muscle growth in gastrocnemius, tibialis anterior and triceps muscles, an increase in the number and cross-sectional area of ventral horn cells and an amelioration of overall motor performances and lifespan [89]. SMN protein levels in the spinal cord and muscles did not change [89]. This finding may imply that follistatin exerts its effect in an SMN-independent manner.

However, later studies using transgenic inactivation of myostatin or transgenic overexpression of follistatin did not confirm these positive results. Genetic knockout of myostatin in a SMA mouse model did not exert a significant impact on muscle development or SMA phenotype in pups [90]. Similarly, SMN Delta7 mice with transgenic overexpression of follistatin showed little increase in muscle mass and no improvement in motor function or survival [91]. A third strategy involved postnatal treatment of SMN Delta7 mice with soluble activin receptor IIB (ActRIIB-Fc) [91]. These mice displayed only minimal improvement in motor function and no increase in lifespan compared to sham controls. A potential explanation for these discouraging results might lie in the severe phenotype of the SMN Delta7 mouse model. Their limited survival (approximately 2 weeks) might indeed be too short for the drug to exert its action. In addition, the SMN Delta7 mouse presents cardiac features that are not observed in human disease [92], and therapeutic strategies acting only on skeletal muscles and not on heart function may fail to prolong survival in these mice. Overall, albeit able to recapitulate features of the severe form of human disease, this model may not represent well type 2 and type 3 SMA patients, the preferred targets of muscle-enhancing drugs.

Some authors attempted to solve this issue by testing myostatin inhibition in a milder SMA model [93]. This model was pharmacologically obtained by treating the SMN Delta7 mouse with a suboptimal dose of SMN-upregulating compound, that extended survival into adulthood but only partially rescued phenotype [93]. Myostatin inhibition was achieved by delivering an AAV serotype 1 (AAV1) encoding follistatin (FS344) via intramuscular injection at postnatal day 14 [93]. Muscle weight significantly increased in several hind limb muscles, including the gastrocnemius, tibialis anterior, and extensor digitorum longus [93]. There was an increase also in overall body weight, but no benefit in survival. These findings suggest that muscle-enhancing therapeutics may work better if used in conjunction with SMN-upregulating molecules [93].

Another rodent model of milder SMA phenotypes, the SMA C/C mouse, that harbors four relative copies of *SMN2*, was used to test myostatin inhibitors. AAV-mediated gene transfer was exploited to deliver either a protease-resistant myostatin propeptide (dnMstn) or the soluble form of the extracellular domain of the activin receptor type IIB (ActRIIB), both able to block the activin signaling pathway [94]. Both treatments were able to improve muscle mass and function in treated mice at 12 weeks of age, compared to controls. Upon histological analyses, the fast fiber type muscles seemed to show greater preservation than slow muscles, while neurophysiological studies revealed a moderate decrease of motor unit number in the tibialis anterior, which could be due either to motor unit loss or neuromuscular junction transmission failure [94]. Taken altogether, these results indicate that myostatin/activin inhibition represents a potential therapeutic strategy to increase muscle mass and strength, but not neuromuscular junction defects, in the less severe SMA C/C mice. Furthermore, AAV-mediated expression of the myostatin propeptide was used on SMA mice treated with morpholino (PMO25) to increase favorable *SMN2* splicing rates [95]. Newborn SMA mice were treated with a single subcutaneous injection of 40 μg/g (therapeutic dose) or 10 μg/g (low-dose) PMO25 alone or in association with systemic delivery of a single dose of AAV encoding the myostatin propeptide. The authors showed that myostatin inhibition acts synergistically with SMN-restoring antisense oligonucleotide at therapeutic dosage (40 μg/g), increasing body weight, muscle mass, fiber size, motor function and physical performance. Mice treated with low-dose PMO25 (10 μg/g), displayed prolonged survival, improved neuromuscular junction maturation and innervation, increased size of sensory neurons in dorsal root ganglia and preservation of proprioceptive synapses in the spinal cord. These data suggest that myostatin inhibition, in addition to the well-known effect on muscle mass, could also positively influence the sensory neural circuits that may enhance MNs function [95].

The prodomain of myostatin shows a low sequence homology with other TGFβ-related growth factors. As a consequence, the use of highly specific antibodies targeting the proforms of myostatin was attempted to reduce aspecific binding to other factors. For instance, the aforementioned SRK-015P is a monoclonal antibody which binds to both pro- and latent myostatin and inhibits tolloid-mediated cleavage of latent myostatin, without binding to mature myostatin nor to any form of GDF11, activin A or other TGFβ family members [96]. Treatment with SRK-015P proved effective in increasing muscle mass and strength, and in preventing dexamethasone-induced muscle atrophy in healthy mice [96]. In another study, treatment with muSRK-015P (SRK-015P with a mouse IgG1 framework to reduce the potential for immunogenicity) improved muscle mass and function and bone tissue structure in two variants of the SMN Delta7 model, which was pharmacologically modified to rescue SMN deficiency either at day 1 or day 24 (corresponding to early or late therapeutic intervention, respectively) [97]. These results point out that specific blockade of myostatin activation could hold a therapeutic potential for SMA. In this scenario, an optimized version of SRK-015P, namely, SRK-015, is currently undergoing clinical testing for SMA under the name of Apitegromab, as discussed below.

### Amyotrophic lateral sclerosis (ALS)

Myostatin inhibition has been evaluated also in preclinical models of ALS, more specifically in SOD1 (G93A) transgenic rodent models. In one study, early treatment with an anti-myostatin antibody produced an increase in muscle mass and strength prior to disease onset and during early stages of disease [98]. During the late stages, only diaphragm muscle displayed a significant difference between treated animals and controls, while there was no difference in time of disease onset and survival. Another group delivered an AAV-encoded follistatin construct to inhibit myostatin signaling. The authors observed sustained increase in muscle mass, myofiber number, and fiber diameter [99]. Likewise, treatment did not affect survival. Subsequently, other researchers attempted to treat SOD1 mice with the soluble activin receptor type IIB (ActRIIB.mFc), showing an increase in body weight and grip strength, an increase in muscle mass and a delay in the onset of weakness whether initiated pre-symptomatically or after symptom onset [100]. However, it did not increase survival nor neuromuscular junction innervation. Given these promising results, Li and colleagues tested a compound (ActRIIB:ALK4-Fc) formed by the extracellular domains of activin-like kinase 4 (ALK4) and activin receptor type IIB (ActRIIB) [101]. When administered to mice, it produced a systemic increase in muscle mass and function and, notably, improved neuromuscular junction abnormalities. Although these studies are not numerous enough to draw definite conclusions on myostatin inhibition in ALS, these treatments were clearly effective in ameliorating motor symptoms but did not affect symptom onset or survival. This may suggest that myostatin-inhibiting treatments should be best used as symptomatic treatments, rather than disease-modifying ones.

## Strategies to modulate myostatin and their applications in MNDs

### Strategies for myostatin inhibition

Since the discovery of myostatin as a critical regulator of skeletal muscle mass, research has focused on the understanding of its molecular and cellular modulators. The long-term goal is the development of treatment strategies that could block myostatin signaling to counteract muscle atrophy. Several progresses have been made towards this direction, with the identification of key molecular regulators of the myostatin pathway. Moreover, several myostatin modulators have already reached clinical trial phase for a broad range of indications, including muscular dystrophy, sporadic inclusion body myositis (IBM), cachexia, aging-related muscle atrophy, obesity, type 2 diabetes, and SMA [102–113].

To date, at least nine biotech and pharmaceutical companies have developed myostatin inhibitors. The approaches that have been tested to inhibit myostatin and its signaling pathway include drugs (small molecules and antibodies) directed against myostatin or myostatin receptors, ligand traps, and overexpression of antagonists, such as follistatin. Up to now, the inhibitors evaluated in clinical trials have fallen into two general classes. The first one (MYO-029, domagrozumab, LY2495655, REGN1033, AMG-745/PINTA-745, BMS-986089/RO7239361, SRK-015) groups compounds that are relatively specific for myostatin, despite some of them present a small degree of cross-reactivity with the protein GDF-11. Conversely, drugs belonging to the second class (bimagrumab, ACE-031/ ramatercept, ACE-083) have a broader range of ligand specificity and are capable of blocking not just myostatin and GDF-11, but also activin A.

### Insights from clinical trials

So far, myostatin inhibition has been tested in a range of diverse conditions, all sharing a known dysregulation in myostatin-relevant pathways. Given its involvement in muscle homeostasis, several trials tested myostatin inhibitors in a range of different neuromuscular conditions (Table 1). Taking a look at the outcomes of the trials, it emerges that targeting myostatin signaling pathway in humans led to inconsistent increase in muscle mass and strength. Some trials described an increase in thigh muscle volume at Magnetic Resonance Imaging (MRI) between 5 and 9% and improvement in the 6-min walking test [112, 114], while other studies did not witness an amelioration in these parameters [103, 107, 113, 115]. Nonetheless, these effects, though significant, were substantially lower than those seen in mice, in which muscle mass increase spanned between 10 to 30% in case of myostatin-specific compounds [97, 116–118].

**Table 1:** Clinical trials testing anti-myostatin antibodies in neuromuscular disorders

Intervention	Comp	Company name	TID/MoA	Trial phase	Disease	Patients enrolled	Inclusion criteria	Delivery route	Primary outcome	Main result	Current stage	Clinical trial number	Ref
RO7204239 (GYM329)	Anti-MSTN mAb	Hoffmann-La Roche	Binding of pro-MSTN	II-III	SMA	NA (est. 180)	Symptomatic, genetic diagnosis of 5q SMA, 2–10 years, ambulant, risdiplam treatment	SUB	Safety, tolerability, pharmacokinetics, pharmacodynamics, efficacy	NA	Not yet recruiting	NCT05115110	NA
SRK-015 (apitegromab)	Anti-propeptide mAb (IgG4/ lambda isotype)	Scholar Rock, Inc	Binding of pro-MSTN	II	SMA	58	5–21 (Cohorts 1–2) and ≥ 2 (Cohort 3) years, genetic diagnosis of 5q SMA, ambulant or non-ambulant but able to sit independently	IV	Efficacy	NA	Active, not recruiting	NCT03921528	NA
SRK-015 (apitegromab)	Anti-propeptide mAb (IgG4/ lambda isotype)	Scholar Rock, Inc	Binding of pro-MSTN	III	SMA	204	2–21 years, genetic diagnosis of 5q SMA, diagnosis of later-onset SMA before receiving nusinersen or risdiplam, non-ambulant, having received nusinersen for 10 months or risdiplam for 6 months before screening, HFMSE ≥ 10 and ≤ 45	IV	Efficacy, safety	NA	Not yet recruiting	NCT05156320	NA
PF-06252616 (domagrozumab)	Anti-MSTN mAb	Pfizer	Binding of ActRII	II	DMD	121	6–15 years, diagnosis of DMD, ambulant, stable glucocorticoid treatment	IV	Safety, pharmacokinetics, pharmacodynamics, efficacy	Treatment-emergent AEs: 115 Serious AEs: 6 (not drug-related) No change in 4SC time from baseline	Terminated	NCT02310763	Wagner et al., [104, 111]
PF-06252616 (domagrozumab)	Anti-MSTN mAb	Pfizer	Binding of ActRII	II	DMD	59	6–18 years, diagnosis of DMD, completed study B5161002, GLDH < 20 UI/L, normal hepatic function, normal estimated hepatic iron content on liver MRI	IV	Long term safety, pharmacodynamics, pharmacokinetics, efficacy	Treatment-emergent AEs: 59 Sever AEs: 4 (1 died of fat embolism from tibial fracture) Serious AEs: 8 (not drug-related) No change in 4SC time from baseline	Terminated	NCT02907619	Wagner et al., [104, 111]
RO7239361 (BMS-986089, taldefgrobep alfa)	Anti-MSTN adnectin	Hoffmann-La Roche	Binding of MSTN	I-II	DMD	43	5–10 years, diagnosis of DMD, able to walk without assistance and to walk up 4 stairs in ≤ 8 s, weight ≤ 15 kg, glucocorticoid treatment	SUB	Safety, tolerability, pharmacokinetics	NA	Terminated	NCT02515669	NA
RO7239361 (BMS-986089, taldefgrobep alfa)	Anti-MSTN adnectin	Hoffmann-La Roche	Binding of MSTN	II-III	DMD	166	6–11 years, diagnosis of DMD, able to walk without assistance and to walk up 4 stairs in ≤ 8 s, NSAA 15, weight ≤ 15 kg, glucocorticoid treatment	SUB	Efficacy, safety, tolerability	NA	Terminated	NCT03039686	NA
ACE-031	Decoy receptor (ActRIIB)	Acceleron Pharma Inc	Binding of MSTN	II	DMD	24	≥ 4 years, diagnosis of DMD, able to walk 10 m in < 12 s, corticosteroid therapy for at least 1 year before enrollment; neck flexors MRC ≤ 4^+^/5	SUB	Safety, tolerability, pharmacokinetics, pharmacodynamics, efficacy	Treatment-emergent AEs: 7 No serious AEs Trend for improvement in the 6MWT distance in the treatment groups	Terminated	NCT01099761; NCT01239758	Campbell et al., [114]
MYO-029	Anti-MSTN mAb	Wyeth (Pfizer)	Binding of MSTN	I-II	BMD, FSHD, LGMD	116	≥ 18 years, diagnosis of BMD, FSHD or LGMD (2A, 2B, 2C, 2D, 2E, 2I), able to walk without assistance, MRC ≥ 3 − and ≤ 4 + in at least 8 of 16 muscle groups, FVC ≥ 60% of the predicted value, ejection fraction > 40%	IV	Safety, tolerability, efficacy	Treatment-emergent AEs: 104 (only accidental injury significantly higher in the treatment groups) No improvement in muscle strength	Completed	NCT00104078	Wagner et al., [103]
PF-06252616 (domagrozumab)	Anti-MSTN mAb	Kathryn Wagner (and Pfizer)	Binding of ActRII	Ib-II	LGMD-2I	19	18–99 years, diagnosis of LGMD2I, able to walk, run, rise from chair, normal hepatic and renal function, normal estimated hepatic iron content on liver MRI	IV	Safety, tolerability, pharmacokinetics, pharmacodynamics, efficacy	NA	Completed	NCT02841267	NA
Bimagrumab (BYM338)	Anti-receptor mAb	Novartis Pharmaceuticals	Binding of ActRII	II	IBM	14	40–80 years, diagnosis of IBM, able to walk ≥ 3 m without assistance	IV	Safety, tolerability, efficacy	Treatment-emergent AEs: 13 No serious AEs Increased thigh muscle volume, LBM, improvement in 6MWT distance in treatment group	Completed	NCT01423110	Amato et al., [112]
Bimagrumab (BYM338)	Anti-receptor mAb	Novartis Pharmaceuticals	Binding of ActRII	II-III	IBM	251	36–85 years, diagnosis of IBM, able to walk without assistance	IV	Safety, tolerability, efficacy	Treatment-emergent AEs: 250 Serious AEs: 72 Deaths: 2 (not treatment-related) No change in 6MWT distance	Completed	NCT01925209	Hanna et al., [107]
Bimagrumab (BYM338)	Anti-receptor mAb	Novartis Pharmaceuticals	Binding of ActRII	II-III	IBM	10	40–75 years, diagnosis of IBM, participation in the CBYM338X2205 study	IV	Safety, tolerability, efficacy	Treatment-emergent AEs: 10 (3 requiring withdrawn, but not drug-related) Serious AEs: 10 Decline of 6MWT distance from baseline	Completed	NCT02250443	Sivakumar et al., [115]
Bimagrumab (BYM338)	Anti-receptor mAb	Novartis Pharmaceuticals	Binding of ActRII	III	IBM	211	≥ 36 years, diagnosis of IBM, participation in the CBYM338X2205 study	IV	Safety, tolerability, long-term efficacy	Treatment-emergent AEs: 191 Serious AEs: 37 Decline of 6MWT distance from baseline	Completed	NCT02573467	Amato et al., [113]

*MSTN* myostatin; mAb: monoclonal antibody; *Comp* compound; *est.* estimated; *SMA* spinal muscular atrophy; *DMD* Duchenne muscular dystrophy; *BMD* Becker muscular dystrophy; *FSHD* facioscapulohumeral dystrophy; *LGMD-2I* limb girdle muscle dystrophy type 2I; *IBM* inclusion body myositis; *Ig* immunoglobulin; *FVC* forced vital capacity; *MRI* magnetic resonance imaging; *HFMSE* Hammersmith Functional Motor Scale Expanded; *NSAA* North Star Ambulatory Assessment; *4SC* 4-stair climb; *MRC* Medical Research Council; *AEs* adverse events; *LBM* lean body mass; *NA* not available; *Ref* reference; *SUB* subcutaneous; *IV* intravenous; *ActRII* activin receptor type II

Myostatin inhibitors were tested also in other conditions characterized by the presence of muscle atrophy. For instance, Novartis tested bimagrumab (a monoclonal antibody directed against ActRIIB) both in IBM, which is characterized by increased Smad signaling in skeletal muscles, and chronic obstructive pulmonary disease (COPD), where myostatin upregulation in skeletal muscles is described [107, 119–121]. However, both trials failed to meet their endpoints [107, 119]. Similarly, myostatin overexpression was implicated in cachexia development in both mice and humans, and myostatin blockade was able to preserve muscle mass and increase survival in cachexia mouse models [38, 122–125]. Nonetheless, these findings did not translate into similar results in clinical trials, as Eli Lilly’s trial of LY2495655 in patients with pancreatic cancer was not able to extend their survival [102]. It must be noted, however, that LY2495655 does not target activin A. Another condition whereby upregulation of myostatin signaling may be involved is glucocorticoid-induced muscle atrophy. Glucocorticoids were able to upregulate myostatin expression through the glucocorticoid response element present in the *MSTN* promoter [126, 127]. Likewise, myostatin inhibition proved effective in halting muscle atrophy following steroid administration in mice [116, 117, 128]. So far, no clinical trial was performed to specifically target glucocorticoid-induced muscle atrophy with myostatin inhibitors. Nevertheless, the muscular dystrophy trials included many patients that were taking steroids as part of their routine clinical care; as mentioned above, none of those trials demonstrated clinical benefit. Dysregulation in these pathways has been associated also to degenerative heart conditions, namely, worsening heart failure and poor outcome after surgical treatment for aortic stenosis [55, 129]. Inhibition of these pathways had protective effects in mice. In addition to that, selective ablation of myocardial-produced myostatin in heart failure mouse models blocked the development of muscle wasting [129, 130]. In humans, myostatin activation was detected in left ventricular tissue samples collected from patients with heart failure who underwent left ventricular assist device implantation [131].

In animal studies, the efficacy of myostatin inhibition was greater in case of molecules capable of blocking both myostatin and activin A [97, 116–118]. Similarly, human trials pointed out that the magnitude of the response seemed higher with biologics with a broader target specificity. For instance, bimagrumab or ACE-031 (a ligand trap) treatment was associated with a 5% to 9% increase in thigh muscle volume, compared with 3% to 5% increase seen after treatment with more specific compounds [132–134]. Interestingly, healthy adults treated with a single injection of either bimagrumab or ACE-031 showed increased muscle volume over 5% after just 4 weeks [133, 134]. The higher efficacy of this class of drugs is consistent with preclinical evidence that both myostatin and activin A are involved in regulating muscle growth. Furthermore, circulating levels of myostatin and activin A are, respectively, seven- to eightfold lower and three- to fourfold higher in humans compared with mice, suggesting that activin A might exert a more prominent effect in humans compared to mice (13).

In addition to that, the efficacy on motor endpoints in clinical trials has been inconsistent and varied not only according to the drug but also to the patient population. Increased muscle mass did not result in clinically meaningful improvement of muscle strength, functional motor scales (e.g., 6-min walking test, stair climbing time, etc.) or self-reported physical function in the trials conducted on patients with muscular dystrophy [103, 104, 111], sporadic IBM [107, 115], neoplastic cachexia [102] and COPD [119]. Conversely, two of the trials that resulted in clear improvements in motor function targeted the elderly population. In one trial, bimagrumab treatment of individuals aged 65 or older with sarcopenia led to improvement in motor performance in the upper limbs (increased grip strength) and, in a subset of individuals, also in the lower limbs (increased gait speed and 6-min walking distance) [135]. In another trial, treatment with LY2495655 brought a significant improvement in motor performances (stair climbing, time to rise from a chair, and gait speed) in individuals aged 75 or older with a history of falls [105]. Age and sex might indeed act as confounding factors in clinical trials, as circulating levels of myostatin were shown to vary according to these variables, as pointed out in the previous sections.

Targeting the myostatin signaling pathway proved effective also in reducing fat mass, an effect that has been clearly seen in obese individuals with type 2 diabetes treated with bimagrumab. The promising Novartis’s trial of bimagrumab showed decrease in total body fat mass by approximately 20%, increase in lean body mass by 4.4%, and decrease in waist circumference by 9.5 cm [109]. Other clinical trials with bimagrumab [106, 115, 119, 132, 133, 136], LY2495655 [105, 108], AMG-745 [137], and ACE-031 [134] evidenced a reduction in fat mass following myostatin inhibition. These findings were in line with results from mouse studies, where both myostatin knockout and myostatin inhibition proved able to significantly reduce fat accumulation [70, 73, 78, 138]. Myostatin targeting was also effective in improving glucose metabolism in both human and animal studies. Indeed, metabolic profile was ameliorated in both genetic [138] and diet-induced [70] rodent models of obesity and type 2 diabetes. In addition to that, a beneficial effect of bimagrumab on the glycometabolic profile (insulin sensitivity, HbA1C) was observed in obese individuals with type 2 diabetes in two clinical trials [109, 136]. In one of these trials, the reduction in fat mass and the improved glycemic control were apparent only 10 weeks after one single dose [136].

Overall, human trials discussed so far led to disappointing results, and this lack of meaningful clinical impact could be ascribed to different reasons. One possibility lies in the pharmacokinetics differences between animal models and humans, leading to insufficient drug exposure in clinical trials, as pointed out by Singh and colleagues [139]. In addition to that, our understanding of the underlying biological rationale might be incomplete, with unavoidable consequences on the clinical indication of different molecules. For instance, there might be compensatory signaling through other related growth factors regulating muscle mass, such as follistatin, that might explain why less specific compounds display a higher efficacy [140]. Another potential issue could be the reduced levels of the circulating target in the selected pathological conditions [86]. Notably, in some conditions the myostatin pathway was shown to be downregulated. Some authors advocate that certain neuromuscular diseases, such as Duchenne muscular dystrophy (DMD) and congenital myotubular myopathy, might belong to this group, so that these patients may be less responsive to therapeutic myostatin inhibition [86, 88]. On the other hand, other researchers argue that, since most myostatin derives from skeletal muscle, lower circulating levels may be the mere consequence of muscle tissue loss and atrophy [75, 141]. Finally, we might be using inappropriate clinical endpoints, focusing, for instance, on muscle size increase. In this scenario, the relatively small increase in muscle mass seen in humans compared to mice seems to be the main driver of the inconsistency in functional improvements that we witnessed in clinical trials. Further studies are surely warranted to clarify the implications of these findings on the results of clinical studies.

### Clinical development of myostatin modulation in MND patients

As regards MND patients, clinical translation was attempted only in the case of patients with SMA.

So far, two myostatin inhibitors reached clinical trial phase for treatment of SMA. A clinical trial on RO7204239 (GYM329) is about to start, while a clinical trial on apitegromab (SRK-015) is ongoing. Apitegromab is a fully human, monoclonal antibody that binds to human promyostatin and latent myostatin with a high degree of specificity, without binding mature myostatin and other closely related growth factors [142]. As discussed in the preceding section on preclinical studies, apitegromab in combination with an SMN upregulator was able to increase muscle mass and strength in SMA mice [97]. These findings paved the way for clinical translation of apitegromab and its evaluation in SMA patients in the Phase 2 TOPAZ trial (NCT03921528). Before translating apitegromab into clinic, a comprehensive preclinical assessment of its pharmacology, pharmacokinetics, and safety across multiple species was performed. Safety studies conducted on monkeys and rats showed that weekly intravenous administration of apitegromab at high doses achieved sustained serum concentration and target engagement, in absence of tolerability issues and treatment-related adverse events [142]. No detrimental effects were observed on neurodevelopmental, motor, and reproductive outcomes in juvenile rats. Having verified apitegromab’s safety on preclinical mammalian models, a phase 1, double-blind, placebo-controlled study on healthy adult subjects was conducted with the aim of assessing safety, pharmacokinetic and pharmacodynamic parameters, and immunogenicity of single and repeated doses of apitegromab [110]. Subjects were administered either single intravenous doses of apitegromab of 1, 3, 10, 20, 30 mg/kg or placebo, or multiple intravenous doses of apitegromab of 10, 20, 30 mg/kg or placebo. The treatment led to a dose-dependent and sustained increase in serum latent myostatin, was safe and well tolerated. Moreover, subjects did not develop anti-drug antibodies. Following these favorable results, the company—Scholar Rock – moved on to a clinical trial of SRK-015 in patients with SMA type 2 and type 3.

## Perspectives and pitfalls: critical questions for future development

Albeit early clinical trials of myostatin inhibition for several muscle-atrophying diseases yielded disappointing results, recent trials raised new hope for a clinical usefulness of these strategies, especially for SMA. Surely, additional studies are needed to refine existing compounds for human delivery. Three main research questions need to be answered, namely, the suitability of the drug, the appropriateness of the clinical indication and the choice of appropriate outcome measures.

A precise tailoring of the drug for human use is needed to address the first point. One critical issue is the choice of ligands to target. Preclinical studies pointed out that compounds with a broader affinity for other TGF-β family members, in particular activin A, are more effective than myostatin-selective compounds [50, 75, 141]. On the other hand, a broader specificity, as it happens for molecules, such as ActRIIB/Fc, carries a higher risk for off-target effects in tissues other than skeletal muscle. For instance, the Acceleron’s trial tested an analogue of the decoy receptor in patients with DMD, and off-target effects such as epistaxis and telangiectasias were observed [114]. These adverse effects were likely due to the ability of ActRIIB/Fc to inhibit BMP-9 (GDF-2) and BMP-10 action. To achieve a broader specificity without incurring in off-target effects, a potential strategy is the “add-on” method, which consists in adding another inhibitory compound targeting the chosen molecule on top of myostatin inhibition. This strategy was attempted by Regeneron pharmaceuticals with the use of combined myostatin/activin A inhibition [49]. An alternative approach could be termed the “dial-out” method, by starting with a molecule with a broad specificity (e.g., ActRIIB/Fc or follistatin) and then engineering it to remove unwanted interactions with certain ligands, such as BMP-9 and/or BMP-10.

Despite these considerations, it is unrealistic to expect the complete avoidance of all off-target effects, even in the case of a highly specific compound. Myostatin and activin A freely circulate in the bloodstream and their receptors can be expressed in a variety of organs and tissues. These observations are further supported by the evidence that systemic administration of ActRIIB/Fc to mice leads to an improved metabolic state and glucose tolerance, increased bone density and more favorable cardiovascular profile, as we discussed in previous chapters. In addition to that, it was observed that postmenopausal women treated with bimagrumab presented lower FSH levels due to the inhibition of activin signaling in the pituitary gland [143, 144]. Surely, this and other extragonadal effects of FSH inhibition, such as bone and adipose tissue regulation, may be exploited in case of certain clinical indications, such as aging or reduced bone mineralization secondary to other comorbid conditions. The downside is that the same effects that are beneficial in certain classes of patients may be detrimental in other populations.

A further method that could increase the specificity of the compounds is skeletal muscle-restricted delivery. However, intramuscular injection does not appear as a feasible method in humans because of the high number of muscles to target. A novel possibility is represented by selective targeting of specific receptors. Indeed, the two type I receptors, ALK4 and ALK5, seem to have a much greater expression in muscle mass than the two type II receptors, at least in rodent models [75]. Further studies are needed to assess whether this is true in humans as well. If this is the case, we could expect that targeting type I receptors might lead to enhanced effects on muscle mass than seen in current trials.

Another major issue that should be addressed is the selection of appropriate clinical indications, with the aim of increasing the likelihood of a clinically meaningful response. So far, myostatin inhibition has been tested in a range of diverse conditions, all sharing a known dysregulation in myostatin-relevant pathways. However, the upstream and downstream involvement of multiple intracellular pathways warrants a careful selection of the disease to target. For instance, conditions, whereby there is a disruption of the IGF-1 signaling, such as sarcopenia or chronic renal diseases, do not represent optimal indications, because IGF-1 signaling is essential for protein synthesis. Another concern relates to the observation of reduced levels of circulating myostatin in certain neuromuscular diseases, namely, DMD and myotubular myopathy, that might thus reduce the efficacy of this therapeutic strategy. Although this thesis is still debated, it sounds reasonable to assume that the presence of a severe muscle atrophy might make it more difficult to observe significant improvements in clinical trials. Therefore, a proper stratification of disease severity before trial inclusion and subsequently the analysis of results in light of the baseline status of the patients could prove helpful. In this scenario, MNDs seem to be a promising target, and clinical evaluation in SMA patients yielded positive results so far. If these promises will continue to be fulfilled, it will be necessary to establish whether they indicate that SMA is particularly susceptible to myostatin targeting or whether they stem from the favorable biological profile of apitegromab. Another interesting target is represented by hereditary IBM, such as GNE myopathy [145]. It was observed that hereditary IBM syndromes might underlie cellular mechanisms related to sarcopenia and aging [145]. Indeed, sarcopenia-related susceptibility genes and related proteins were shown to interact with GNE and other components of the myofibrillar apparatus, impacting muscle endurance and stability [146]. Because of these findings, GNE myopathy might benefit from the use of myostatin-targeting therapies, even though no clinical trial has been conducted yet.

We also need to take into consideration the choice of measures of efficacy. In some trials, actual muscle strength was used to this aim. However, other chosen parameters may not directly track muscle mass or strength. It stands out that a major challenge to overcome in future trials will be the definition of appropriate functional endpoints that may accurately capture the beneficial effects of myostatin inhibition. Despite all the trials conducted so far focused mainly on muscle size and motor performances, studies in animals and humans revealed the importance of myostatin pathway modulation on metabolic parameters, such as glucose homeostasis and body composition, as well. Researchers will need to devote more attention to this aspect to improve the design of clinical trials and to widen existing indications.

## Conclusions

MNDs represent a huge burden for affected patients, their caregivers, and society in its whole. Despite this unfavorable scenario, several advancements regarding knowledge of pathogenic mechanisms and therapeutic strategies have been made in the past years. It has also become evident that traditional neurocentric theories that considered SMA and ALS to be cell-autonomous diseases are now obsolete; indeed, a wide range of cells and systems have been shown to participate in the pathogenic and degenerative process [10, 147, 148]. Skeletal muscle tissue is particularly interesting from this point of view, being one of the major victims of motor neuron degeneration, but in spite of that, very few therapies aimed at restoring skeletal muscle trophism in MNDs have been investigated so far. Luckily, several research groups and pharmaceutical companies have now recognized the potential of these therapeutic approaches for a wide range of indications. Insights provided by clinical and preclinical trials demonstrated that muscle-enhancing therapeutics, such as myostatin inhibitors, were able to exert beneficial effects in ALS and SMA models and in SMA patients. In the current landscape of available treatments, myostatin inhibitors could prove particularly beneficial for SMA patients who do not respond or cannot use SMN-upregulator therapies. Moreover, the combination of SMN upregulation and myostatin inhibitors might maximize benefit for SMA patients by acting on several molecular levels. In the case of ALS patients, no disease-modifying treatment is currently available, with the notable exception of tofersen for *SOD1* ALS patients. Because of that, the availability of therapeutic weapons able to improve strength and slow down the loss of motor abilities would answer to a huge unmet need. Overall, the inhibition of myostatin and its related pathways raises hopes for a better care of MNDs in the near future.

## Data Availability

Not applicable.
